# A histone point mutation that switches on autophagy

**DOI:** 10.4161/auto.28767

**Published:** 2014-04-24

**Authors:** Tobias Eisenberg, Sabrina Schroeder, Sabrina Büttner, Didac Carmona-Gutierrez, Tobias Pendl, Aleksandra Andryushkova, Guillermo Mariño, Federico Pietrocola, Alexandra Harger, Andreas Zimmermann, Christoph Magnes, Frank Sinner, Simon Sedej, Thomas R Pieber, Jörn Dengjel, Stephan Sigrist, Guido Kroemer, Frank Madeo

**Affiliations:** 1 Institute of Molecular Biosciences; University of Graz; Graz, Austria; 2 INSERM U848; Pavillon de Recherche 1; Villejuif, France; 3 Metabolomics and Cell Biology Platforms; Institut Gustave Roussy; Pavillon de Recherche 1; Villejuif, France; 4 Université Paris Sud, Faculté de Médecine; Le Kremlin Bicêtre; Paris, France; 5 Division of Endocrinology and Metabolism; Department of Internal Medicine; Medical University of Graz; Graz, Austria; 6 HEALTH—Institute for Biomedicine and Health Sciences; Joanneum Research Forschungsgesellschaft m.b.H.; Graz, Austria; 7 Department of Cardiology; Medical University of Graz; Graz, Austria; 8 Department of Dermatology; University Freiburg Medical Center; Freiburg, Germany; 9 Institute for Biology/Genetics; Freie Universität; Berlin, Germany; 10 NeuroCure, Charité; Berlin, Germany; 11 Equipe 11 Labellisée Ligue Contre le Cancer; INSERM U1138; Centre de Recherche des Cordeliers; Paris, France; 12 Pôle de Biologie; Hôpital Européen Georges Pompidou; AP-HP; Paris, France; 13 Université Paris Descartes; Sorbonne Paris Cité; Paris, France

**Keywords:** acetyl-coenzyme A, aging, ATG, autophagy, epigenetic, histone acetylation, transcription

## Abstract

The multifaceted process of aging inevitably leads to disturbances in cellular metabolism and protein homeostasis. To meet this challenge, cells make use of autophagy, which is probably one of the most important pathways preserving cellular protection under stressful conditions. Thus, efficient autophagic flux is required for healthy aging in many if not all eukaryotic organisms. The regulation of autophagy itself is affected by changing metabolic conditions, but the precise metabolic circuitries are poorly understood. Recently, we found that the nucleocytosolic pool of acetyl-coenzyme A (AcCoA) functions as a major and dominant suppressor of cytoprotective autophagy during aging. Here, we propose an epigenetic mechanism for AcCoA-mediated autophagy suppression that causally involves the regulation of histone acetylation and changes in the autophagy-relevant transcriptome.

Besides its role as a fuel for several anabolic pathways, the central energy metabolite AcCoA represents the only known donor of acetyl groups for protein acetylation. This function has lately been recognized as an important regulatory instrument, affecting enzyme activities, and protein complex stability as well as the epigenetic status of chromatin and transcription factor activity. Among others, the autophagic pathway has repeatedly been reported to be subject to regulation by various protein acetylation and deacetylation activities. Therefore, we reasoned that AcCoA availability might directly modulate autophagic activity. Indeed, using the established model of yeast chronological aging, we recently unveiled that the nucleocytosolic pool of AcCoA functions as a metabolic repressor of autophagy in aging cells. Depleting the nucleocytosolic pool of AcCoA is sufficient to induce and maintain high autophagic rates crucial for longevity. This mechanism is phylogenetically conserved, since brain-specific knockdown of nucleocytosolic *Drosophila AcCoA synthetase* enhances autophagic protein clearance and prolongs life span.

In yeast, AcCoA is synthesized in the mitochondrial and the nucleocytosolic compartments, generating 2 distinct subcellular pools. This enabled us to spatially dissect the role of AcCoA biosynthesis pathways in regulating autophagy. Blocking the mitochondrial route to AcCoA by deletion of either the yeast CoA-transferase-encoding gene *ACH1* (forming AcCoA from acetate) or the gene for the mitochondrial pyruvate transporter, *FMP37/MPC1* (shuttling pyruvate into mitochondria for subsequent conversion to AcCoA) caused a shut-off in autophagic flux upon aging. Both genetic constraints correlate with cytosolic accumulation of the AcCoA precursor acetate. This leads to hyperactivation of the nucleocytosolic AcCoA-synthetase Acs2, culminating in increased acetylation of cellular proteins, particularly histones. Acs2 activity is causally responsible for autophagy limitation since simultaneous knockdown of *ACS2* recovers autophagy in *ach1* mutant cells—albeit acetate accumulation still occurs.

Importantly, knockdown of *ACS2* not only reinstates autophagy but also completely prevents histone hyperacetylation induced by deletion of *ACH1*. We thus hypothesized that epigenetic changes (i.e., modulation of histone acetylation) guide autophagy in the long-term context of chronological aging, mediating the cellular adaptation to continuous stress. In line with this idea, histone H3 hyperacetylation, resulting from an Acs2-mediated increase in nucleocytosolic AcCoA biosynthesis, is associated with transcriptional downregulation of several autophagy-essential *ATG* genes (*ATG5, ATG7, ATG14*). This consequently leads to reduced protein levels (as shown for Atg7), suggesting that limitation of essential *ATG* transcripts may restrict age-associated autophagic activities. However, complex changes of the autophagy-relevant transcriptome are likely to be present under these conditions and the subset of genes that accounts for AcCoA-mediated repression of autophagy must be elucidated in the future.

To demonstrate the causal involvement of post-translational modifications (PTMs) at histone lysyl residues in regulating autophagy during aging, we introduced point mutations at those histone H3 sites that we had found to be hyperacetylated upon increased nucleocytosolic AcCoA production. As the precise stoichiometry of histone acetylation is currently not described for aging yeast cells, we created a panel of (nonacetylable) lysine mutations that likely mimicked differently acetylated states compared with wild-type conditions. Therefore, unique deacetylation-mimicking lysine to arginine (KR) mutations were also combined with lysine to glutamine (KQ) mutations, to simulate various degrees of acetylation “locked” at a certain level. Among the tested mutants, intriguingly, a mixed mutation of *H3-K14,18Q/K14,18R* allowed normal growth but increased the age-associated autophagic activity above that of wild-type cells (Fig. 1). While point mutations per se fail to completely mimic the highly refined, time- and location-dependent changes of PTMs that occur in chromatin in vivo, this finding demonstrates the basal capability of histone PTMs to modulate autophagic activities during the process of aging. The precise mechanism through which epigenetic changes translate to the autophagy-relevant transcriptome remains to be addressed and may well include a crosstalk with specific transcription factors (Fig. 1).

**Figure:**
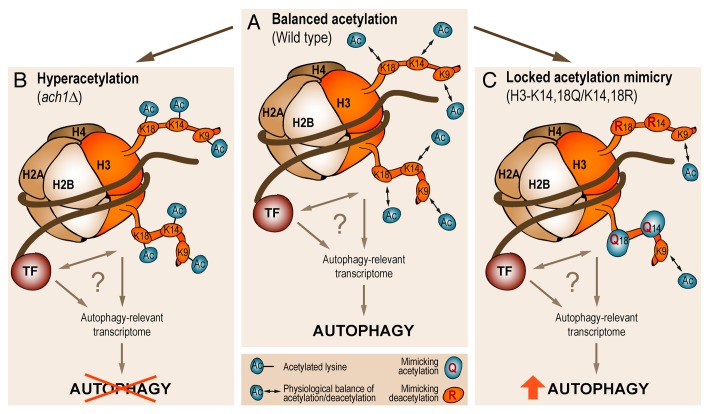
**Figure 1.** Epigenetic changes in histone acetylation determine autophagy in the long-term context of aging. Schematic model depicting the consequences of histone H3 acetylation in age-associated autophagy. (**A**) While dynamic acetylation at a balanced physiological level permits wild-type cells to adapt with autophagy to aging conditions, histone H3 hyperacetylation (**B**) as a result of Acs2-hyperactivity and increased nucleocytosolic acetyl-coenzyme A production is associated with a loss of autophagy during aging. (**C**) Partially “locking” the epigenetic status of chromatin by a combination of lysine to glutamine (KQ) as well as lysine to arginine (KR) mutations at the indicated H3 lysyl sites, thereby mimicking a defined (de)acetylation status, ameliorates the autophagic response to aging. The question mark underscores uncertainties about the precise mechanism of how chromatin modifications regulate the autophagy-relevant transcriptome as well as about the precise set of affected genes. The activity of chromatin modifying proteins (and protein complexes), including transcription factors (TF), may assist in translating altered histone acetylation to autophagy regulation.

Other recent studies reported on the epigenetic modulation of autophagy. Upon modulation of histone H4 acetylation at lysine 16, cells responded by changing their autophagy-relevant transcriptome that determines whether autophagy becomes a vital or lethal process, demonstrating once more that (nuclear) epigenetic events may have been underestimated in their capacity to fine-tune autophagic activities. This may particularly apply to conditions that differ from the most frequently studied case of autophagy induced by acute nutrient withdrawal. Such “tuning” capacities reportedly relate to the general autophagic flux, the frequency of cells with active autophagy, the vital or lethal outcome of autophagy, and the size of autophagosomes. It is tempting to speculate that epigenetic control of the autophagy-relevant transcriptome would also influence the spatiotemporal specificity of cargo selection during macroautophagy.

AcCoA integrates vital nutrition pathways and thus translates environmental clues to the epigenome. Therefore, our findings may explain diet-dependent life span and autophagy regulation, and could extend to other pathophysiological conditions known to depend on the status of histone (de)acetylation. Pharmacological interventions targeting the nucleocytosolic pathway of AcCoA generation may represent an attractive strategy to combat autophagy-associated pathological conditions.

## Notes

EisenbergTSchroederSAndryushkovaAPendlTKüttnerVBhukelAMariñoGPietrocolaFHargerAZimmermannA Nucleocytosolic depletion of the energy metabolite acetyl-coenzyme a stimulates autophagy and prolongs lifespan Cell Metab 2014 19 431 44 10.1016/j.cmet.2014.02.010

